# Microdesign using frictional, hooked, attachment mechanisms: a biomimetic study of natural attachment mechanisms—Part 3

**DOI:** 10.1186/s40638-016-0040-4

**Published:** 2016-05-05

**Authors:** Bruce E. Saunders

**Affiliations:** University of Bath, 54 Ballance Street, Bath, UK

**Keywords:** Microadhesion, Miniaturisation, Cellulose, Chitin, Hook, Self-assembly, Scaling effects, Biomimetic, Bioinspire, Probabilistic, Friction, Fields, *Arctium minus*, *Apis mellifera*, *Omocestus viridulus*, Biosensor, Dimensionless groups, Copper

## Abstract

Part 1 completed the studies of five long-shafted, cellulose, frictional, hooked probabilistic fasteners. Part 2 identified three substructures prevalent in the natural world for probabilistic fasteners and detailed the collection of voxel dataclouds while measuring from the natural fluorescence of their composing chitin and cellulose under the laser illumination of a confocal microscope. In this part 3, consideration is given to the development of a behaviour-optimised bioinspired probabilistic attachment system that is thermodynamically inert due to attachment substructures, such as interlocking setae, that act as arrestors and temporary interlocking devices. The three devices of part 2 are considered for their relative merits, and one part is modelled for a rapid prototyping device. If one is considering the question of shape versus material, then it is at this stage that it is a very important issue since one is considering fundamental, simple shapes and the materials used to form them are of finite variety. Hence, the final design will hinge upon design for manufacture and component material qualities, in this case copper.

## Background

It is strange to think that one can find a biomimetic principle on demand. This study, however, has found at least one in the consideration of its objective, to take all known studies of hooks and to compare them and others in order to define a new hook that is advantageous to design for a purpose that precludes all known uses so far, i.e. that is intended for a use that has not so far been defined. However, it is such that it can be assumed that all the new designs are going to be on the purpose/possibility frontier, namely in this case, microdesign and the design of micron-ranged size structures that do not altogether behave in a “normal” way under use, such as the tarsal hooks of an insect and their manner of sticking or adhering to a surface. It is somewhat of a mystery yet but which can be considered and mimicked to find its own properties that reflect their sizes (the hooks).

It is through the design of these autonomous structures and their variants that microdesign such as those parts used by computer-aided medical devices are possible. But it is a mystery yet in the scientific literature, how these marvels are designed such that it can be studied. There are new ways of doing things that need chronicling so that their progress can be chartered and modified according to new discovery.

The title of the thesis proposal was “The Functional Ecology and Mechanical Properties of Biological Hooks in Nature”. This tells us that each hook must be studied as a part of a system; hence, the complete set of mechanical properties will only be revealed once the hook has been brought into interaction with a substrate of some properties of its own. Attention is now drawn to the publication of a paper that is significant to this study, namely [1] that is recently published where in it is described how a process of electro-chemical deposition is used to draw the bead of the new substance, namely copper, to draw in the “cubic pixels” or voxels, that then make up the shape required or drawn, or in this case, scanned on a confocal microscope as executed in paper 2 of this research upon three specimens which were all measured under the microscope [2]. This enables the process to continue, of evaluating the progress such that it can be concluded how to best design a hook made of copper at the size of 100 micron span say, manufacturing it in a macrofashion and predicting its behaviour in an absolute fashion.

Being considered here is a necessarily obscure manner of approaching the issue, to predict what will be the manner of making these structures and how the design will process the data of the new part such that it will behave in a fashion that will be predictable or not depending on the way it is fashioned or the material itself and its issues with solidarity and maintaining shape. How to draw a free-body diagram of such a structure, for instance, would be quite specific and yet very different from predicted Newtonian behaviour. There is a way to get all of the parts of the design into one sphere and that is to generate a curve that shows their behaviours under different variants, according to different criteria. It is not going to be easy to explain the use of dimensionless groups to produce fluid-flow performance curves, but these make the use of criteria that may come into the design such as Brownian motion or molarity of particles in self-assembly or electro-chemistry. Each will affect the solution of the entropy of the system but will also preclude the discovery of any new criteria unless they are included in the symmetry of the equation that makes all the forces equal at any one state or stage. Otherwise, we will have movement, and this is what we are trying to arrest with a probabilistic fastener. In short, all forces must be balanced.

Saunders [3] describes the discovery of an apparent tensile shape/size-scaling effect while completing a study of long-shafted cellulose hooks started by Gorb. The *Arctium minus* hook was found to be exceptionally strong, an effect answered by calculation as being an illustration of the hook’s propensity to be strong through either composition or shape and therefore span and fibre content. This is of little importance since we are simply warned that there are these effects present in Nature.

There is however a consequence of our study which is that we have a selection of five long-shafted hooks of cellulose from which to choose and the most frequent and strongest, namely *A. minus* was chosen, as per George de Mestral and his Velcro. It is then that we undertook to scan the hook under the confocal microscope together with two other samples noted for their frequent occurrence in British wildlife, namely the tarsi of the British common wasp and grasshopper (*Apis mellifera* and *Omocestus viridulus*). This led to the conclusion that we had the route to three evolutionary pathways plotted on our confocal microscope and preserved for further analysis or manipulation in the form of .tiff files [2]. Each is a permanent reusable fastener, and yet each does equip its owner without the need for a sample to identify it and make it from, i.e. they are without a pattern, possessing only a genetic code for growth and form.

### Functional ecology

When considering the system of the hook-shaped structure, going back to the Cambrian Era, one is struck by the fact that there is no apparent control system. Natural Laws prevail. The first hook shapes appearing on the fossil record were of primitive cellulose and chitin.

When studying a biological specimen the prescribed ethos is to study its interaction within its system since it is the system that is of interest to a biologist and to a designer. The entire system needs to be considered, of a frictional fastener such as the probabilistic (i.e. non-random) fastener which the hooks *A. minus* form, and how the fastener shall be designed is based upon the performance of these hooks and their substrates. In the case of the growth and formation of hook shapes in Nature, their biochemistry will be of equal importance and must have been fundamental to the very first organisms that appeared with shapes of biomaterial.

It is possible to break up a system into discrete segments or sections, and this enables the identification of biodesign indicators such as scaling effects that predict the alignment of microfibrils for instance or the percentage volume required of a matrix cavity in a gel.

A free-body diagram is such an example and is selected and constructed along subjective lines for the purposes of force analysis. A free-body diagram can enclose an entire system or can be used to analyse part thereof.

### Botanical hook structures and biodesign indicators for a reusable, frictional, silent, probabilistic attachment mechanism

What we need to consider is that we have passed step one, the choice of a cellulose representative of a single-hook fastener type, exposed on the end of a shaft where behaviour is more isolated from the base or surface from which it originates. This led us to a discussion above. There is then the choice of three, which has been presented [2]. Now it must be established which of the three is most suitable for pre-production analysis and study, and a cellulose single hook has been chosen. This is because

- It is a non-assembly.
- It is long and therefore accessible to the head of a 3-D printer for its overhang if necessary.

For this work there are special qualities that may be included in the design which may or may not increase the quality of the attachment such as the inclusion of setae-like protrusions from the shaft or hook itself. These might ease adhesion.

So it is a conclusion of the previous research that we must produce a sample hook in .stl data file format that can be used as a basis or datum structure and that can morph into variations according to need and application. It is then understood that all the variations will be available to research, as would be those of the other two samples which have yet to be deciphered onto Solidworks, but it may not be deemed necessary in the light of [1] where direct control of the data transfer is allowed, and thus, the treatment of the structures under testing will be available in copper only which is a very conducive, malleable substance. From a point of view of application, what needs be considered is the requirements of application of the attachment and what bioprinciple we can derive from this structural mimicry and that includes the obvious—a tactile manner of carrying a load up a direct incline of 90°. It also includes the obvious use of copper’s varying impedance under stress which is used in strain gauges and can now be used in the biomedical sensor field.

It is with this in mind that all thought of concluding with a 3-D representation of all three when they are quite readily available through the microscope’s own image-ware is not economical. Instead, a single sample is included that is to scale and leads to the following development of the design for the purposes of manufacture and testing. It is noted here that all the sundry tests have been carried out through the Solidworks software itself such as FEA, but we do not know the true directions from which forces are applied when considering analysis of the hook. A point load seems inappropriate as does a limited forcing being applied through the length of the shaft in sheer. It should be under pressure throughout, not as it seems here, as the cross-stresses are prevalent throughout the real-life loading of the hook.

The point of using Solidworks, in spite of its problems with an analogous material, is that it converses with a rapid prototyping device and produces a file in .stl format, but its FEA capabilities which are vastly insufficient.

### Aim

To transfer the data from SEM to 3-D digitised form of an *A. minus* long-shafted hook, one of the three specimens selected and measured in [2] so that it can be used as a base design for the morphing of shape into variants for testing, in the understanding that the ultimate bioinspired principle being studied is microadhesion; therefore, the test specimen is at the limits of manufacturing capability. It is to be drawn and then analysed using the latest available software (circa 2004). All information pertaining to this subject area is to be noted.

## Methods

### Morphological recording: 2-D digitising

2-D digitising an object is, as it implies, limited by the effect of distortion by the planar image on the 3-D object. This makes it inaccurate, but for the dimensions being considered it was considered sufficient. In the pursuit of the .stl file and the form of 3-D computer graphics output that could be altered, the final conclusion of [2] was to make a direct reconstruction of a model in Solidworks. To gather the data 2-D digitising was applied. Thereafter, a model was constructed of a field of hooks that could be rapid prototyped and manipulated for testing purposes using the new method outlined in [1]. This is the only way of creating these structures on record. Each sample can now be rapid prototyped in copper with s suitable device and tested for its features as needed. This is for further research.

**Fig. 1:**
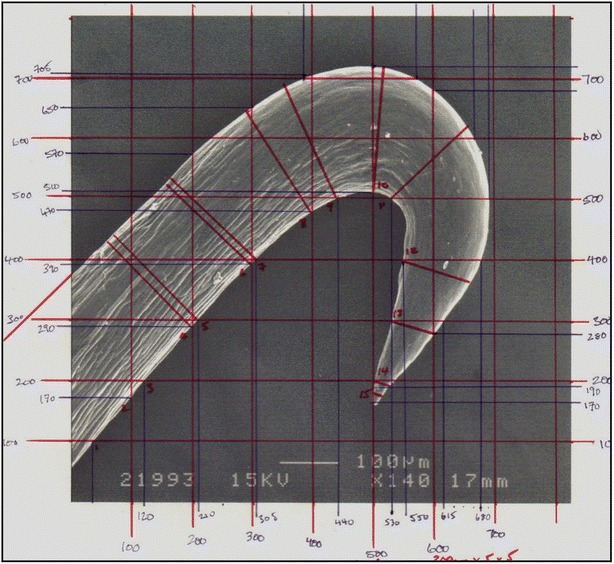
Electron micrograph with grid superimposed, of an *A. minus* hook in profile. Each *bar* represents an interval of 100 microns. This is one of the three species studied in [2] and measured under a confocal microscope set for cellulose then chitin. Both materials fluoresced

**Fig. 2:**
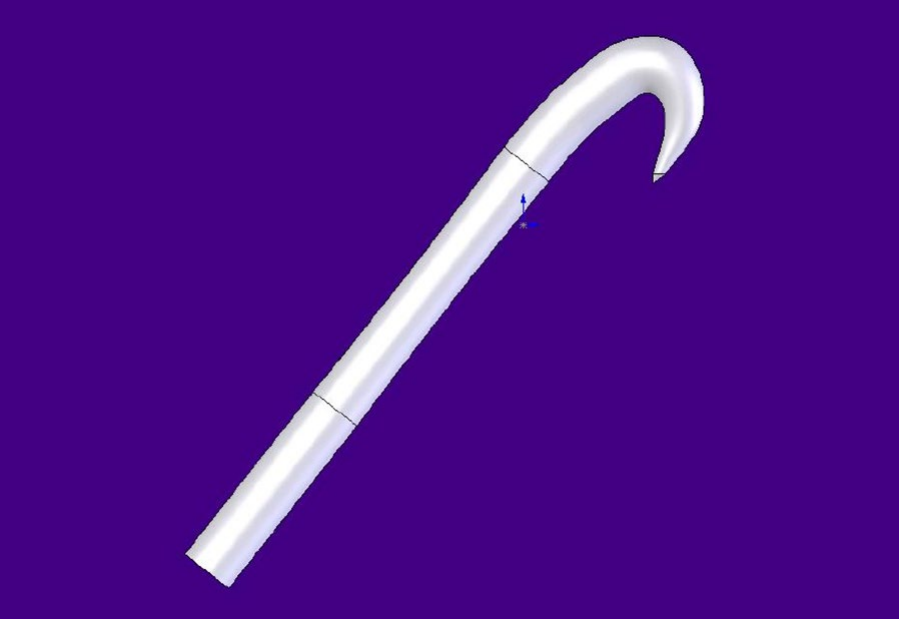
3-D image of reconstructed *A. minus* hook. *Note* the material analogue is uniform and homogenous. Only the hook is an obvious stress concentration. Otherwise, there are no obvious indicators of conservation of material due to lack of applied stress. Hook span = 200 microns

Using Fig. 1 it was possible to digitise two splines for the inner and outer profiles. Diameters were measured perpendicular to the inner spline and used to reconstruct the hook using the loft feature, the result of which is shown below in Fig. 2.

The hook and shaft are drawn in proportion, and it was observed at this point that given Nature’s reputation for energy efficiency and balance, the shaft walls are near parallel, making it clear that they are hiding some secret with regards their form. And that is the inner form, the non-homogenous fibrous, cellular flesh of the hook and shaft and their inner control system or genetic instructions that led the flower, mauve and bright and well suited to the pollinators of the area to transform into the perfect seed dispersal mechanism using the same structures, though desiccated. The flattened flange is optional, but follows from the fact that there are further structural options available, such as flanges or setae, substructures that could play a part in anchoring more firmly in the substrate or providing a brake for adhesion and detachment, mimicking the attachment mechanism of the dragonfly head arrestor mechanism [4]. At this stage a universal foot is being considered for a one-size-fits-all attachment mechanism that can anchor to a wall or vertical flat surface irrespective of the size of the object, and then, it shall be scaled down according to need and ability to adhere and detach repeatedly.

### Finite element analysis

Committing this image to FEA was in fact superfluous but analogous to the execution of the package. The material analogue is very unsuitable. But it illustrates one further point to be made about FEA, and that is it is wildly unsuitable for composite modelling of any form. And further it is known that the cuticle surrounds the cellular content of the hook and this forms a sheaf much like a thin-walled pressure vessel. The shaft resembles a long thin tube, and it deforms under bending like one too. Therefore, the FEA in this package is not advantageous to analysing the cellulose behaviour. However, it is good for measuring the distortion under loading of a micron-sized copper hook, but one must get the loading correct.

### Assembly of the probabilistic, frictional, multiuse, silent, attachment mechanism morphology for a universal foot based upon cladistic studies

This was the first thing that was considered: How to hold the detached figure of the hook between two solid handles to manipulate it when complete. It was decided that it was of no purpose whatsoever to make do with a size-limited sketch and that only the real size specimen and model would do, and therefore, it was a necessary feat to be able to image the specimens completely so as to be able to visualise their capabilities in a virtual reality medium or in a model for an attachment mechanism for manufacture. However, [1] has presented feat of engineering that must be used.

Figure 2 shows the configuration of a test specimen, for a single evolutionary path, that of the *A. minus*. There are two more specimens in part 2 [2] that give the same result, but have not been reproduced in Solidworks for the purposes of economy. These two own the second and third position in the ranking of all possible manufactureable attachment mechanisms, second only by nature of their complexity, one being of two parts, each identical, and the third made up of three parts, two of which are identical. Again with chitin, one wonders whether a thin-walled pressure vessel calculation under bending would be more suitable than the FEA of a homogenous material that Solidworks allows for.

## Results

The standard form of a result table is omitted here because it has yet to be performed. This is a way of getting through to the reader that it is about the morphology or shape of the characteristic attachment mechanism and not its FEA. There are no FEA results although there are indicators on the drawings. This is for the purposes of illustration only.

### Describing the reconstruction from 2-D of the *A. minus* hook

It was once impossible to take this work past the hypothetical allusion to a possible solution through the invention of some material that could be used to manufacture at this scale. This changed directly as a result of [1]. This material is now selected as being copper. It is with a certain use that we associate copper, namely conductivity of electricity and heat, not only that it is “green” material that it is relatively easily reclaimed.

Thus, we are arriving at a solution to the problem of how to attach the smallest of electrodes to a motherboard for instance, using friction. It is not necessary to say more, just to apply the testing array of formulae to show that it is feasible to get to this end solution through the manufacture of these hooks using this method.

As was discussed earlier in the “Background”, it is the choice that matters, of the shape that is most appropriate to suit the purpose as illustrated by Nature. Here we have dictated that it should be the long-shafted hook of the *A. minus* that is the model, the decision made as a basis of the results of part 2 of the study when we discovered that the evolutionary sparkle had gone into the development of a cellulose hook that was fibrous and therefore non-homogenous. Therefore, it can be predicted from shape efficiency constraints that all of the hook will not be used to absorb stress in copper where it is homogenous in structure as laid down by the layered manufacturing device. So it is true that there will be some use in FEA but only if specified correctly for forces and anchorage points and surfaces that are accurate. It will be the same for both of the new acts called the error note of the state, namely the way in which it uses a lot of its energy to absorb sound and noise to stop it from being a friction-destroyed device and rather reusable without destructive tendencies. This will be shown as heat of friction and not available to be used as noise or light as an ignition spark.

**Fig. 3:**
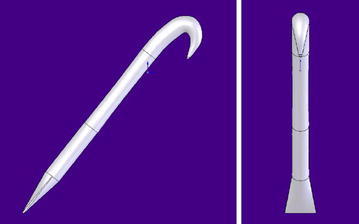
Front and side views of hook with tapered shaft as per the *A. minu*s hook. At this stage we are looking towards a universal probabilistic fastener that can be morphed from this structural example, or one of the other two or a combination or two or more forms/assemblies

**Fig. 4:**
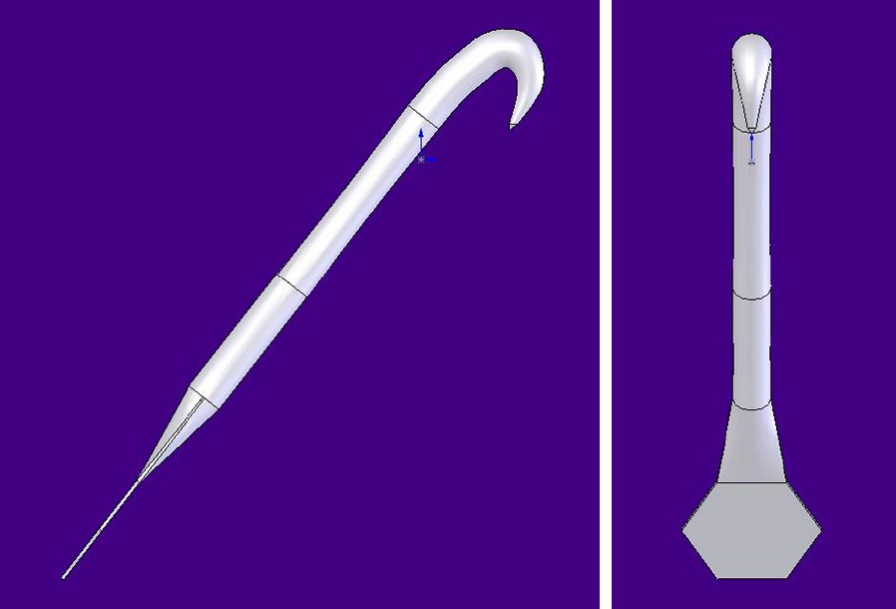
Front and side view of *A. minus* hook with added flat hexagonal flange. Basic assumptions are made here about the forming of the structure. The configuration depends entirely on the configuration of the layered manufacturing device and its pattern of electro-deposition of copper and its jig

Figures 3 and 4 below show the way that the hook was drawn or constructed using the software. It must be done like this for the first principle of the test; thereafter, it is within the possibilities of Nature itself that it will form a new hook in the future when it is able to adapt to the new shape of the substrate. Then, it will adapt through malleability, and thus, a new hook shall be developed that does not forego the use of new materials but rather dictates how they should be formed to have properties to endure and make it feasible.

**Fig. 5:**
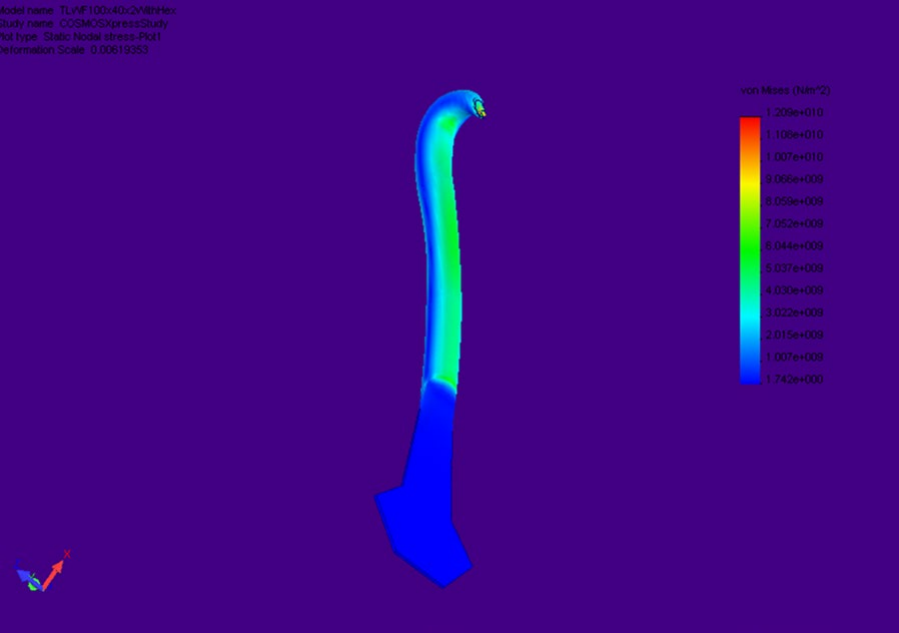
The maximum deformation under loading. A point load at the tip, constrained at the base along the flange. There is nothing unexpected about the mode of deflection

Figure 5 below shows the loading and mode of distortion of the hook, flexing and absorbing the extension and movement of the tip of the hook.

### Hook field structure

It has been noted in the *Nachtigal* [5] that hooks in nature are assembled in different configurations and numbers. Gorb compared the mass of the fruit with the contact separation force of single hook in order to assess the hook performance for each species and the number of hooks required to support the fruit which could be viewed as a measure of design efficiency. This is of course, Nature’s design. Instead, this research continues to avail itself of the known thing that is the scaling effect and the fact that hooks in a field with a modular design could help fulfil a number of roles or permutations and in fact is necessary in order to consider these tiny hooks at all, to consider them collectively.

With the modelling process with a development of a modular hook with a supporting flange analogue, the opportunity exists to experiment with:

1. Field configurations, densities and numbers.
2. Flange shapes, i.e. square, rectangular, circular and octagonal
3. Flange attachment mechanisms for both attaching bracts to each other to form composite fields as well as for attachment to a structure with a further attachment mechanism as a substructure.
4. Flange shapes also offer the opportunity to manufacture the hooks in flattened rows.

**Fig. 6:**
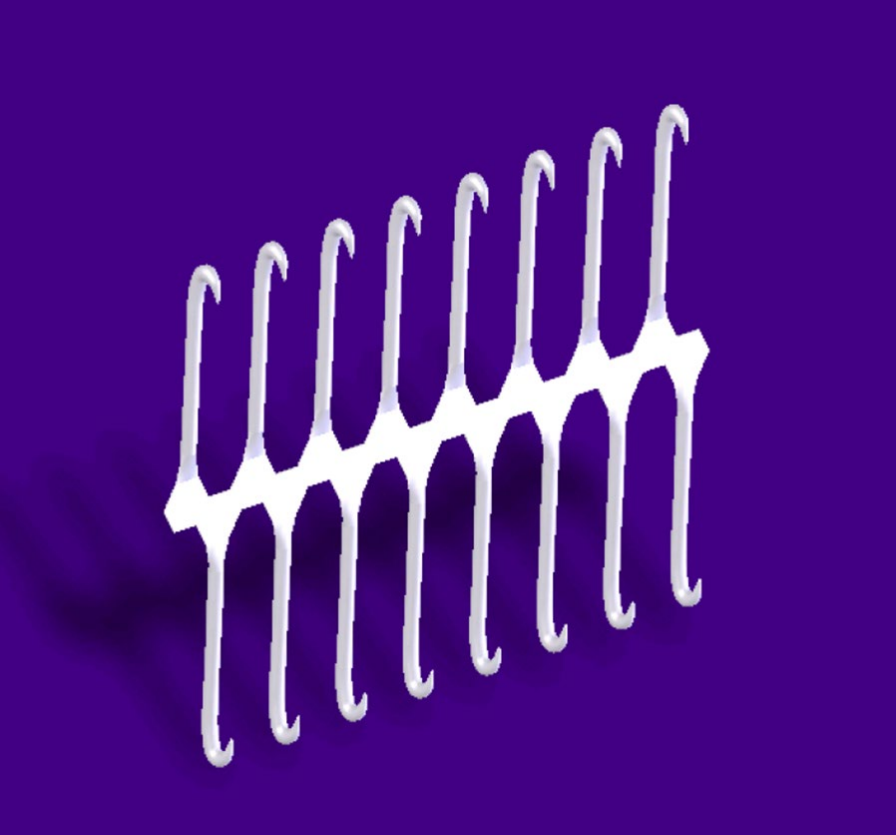
A zipper configuration in isometric view. This illustrates the possibilities of a composite formation of long-shafted hooks acting a coordinated fashion. The point being illustrated here is that although we are seeking a universal “foot”, it is as unlikely to look like a foot as a drone looks like a hummingbird

Attention is drawn to Fig. 6 for extensions to the model:

## Discussion

The 3-D rendering of the model is hoped to be probabilistic, but it is impossible as of yet to produce a model of the scale required using a rapid prototyping device, to produce a product of some variant of the 3-D model as a testable product at the scale to take advantage of the hook-span scaling effect.

The collective effect of the hooks at a small scale in size should be markedly different from that of the arithmetic sum. Under the static load of its own (small) mass it seems it is largely friction that holds the attachment in place, but atomic force microscopy illustrates the strength of atomic forces such as covalent bonding which occurs with the surface molecules as two surfaces approach each other, particularly if they are both hydrophobic such as dry wool and a seasonally desiccated seed pod. Local, internal, turgor pressure will have an effect upon the surface area that forms the bond as will any adhesives, chemical or otherwise. The loss of friction through moisture exposure and thin-film flow must be studied too, for the opportunity to perfect the on–off release mechanism of the foot, short of using a shape memory material.

The desire is for a 3-D computer graphics model of a probabilistic fastener that does not destroy itself or the substrate and that is able to attach and detach to a variety of them and that can be analysed using computational analysis, technologies and techniques. It will be friction based and in the realm where masses and reactions to them are small compared to the frictional and other forces generated.

Walking the line bridging engineer and biologist, with knowledge in an exchange, it should be noted here that the Solidworks software is not of sufficient capacity for the demands of working with a complex biomaterial.

## Conclusion

As part 3 of this series of three papers this culminates from the consideration of the results of research into five species of long shaft hook including the *A. minus* species, various microscopy techniques including confocal microscopy and the design of a bioinspired model for hooked structures of the order of 200 microns span, and two further attachment mechanisms of similar size, of insect chitin.

There are limitations to what can be achieved by the pull of technology. Excited at first by the discovery of the cubic voxel result of part 2 [2], it was soon dampened by the result that it was impossible to work with the voxels in C++. It is hoped that this will yield further work until a proper procedure is established for the study of these attachment mechanisms at their natural scale and order of size in various materials.

The 3-D model produced in Solidworks 2004 is suitable for application to fields of long-shafted hooks at a micron-size, and its qualities have to be assessed in a testing/manufacturing circumstance. It is expected that these attachments will be probabilistic and frictional in behaviour. The rate of change will vary from hook to hook as the size/shape changes, and this can only effectively be measured using the new way of manufacturing the hooks through electro-deposition such that they are of the size but of a different material, thereby mimicking the scaling effects and the biological principal that guides their behaviours governing the size/volume, strength/weight, friction coefficient (*μ*)/size and other relations. It must be remembered that the scaling effects associated with a burdock hook may not be related directly to Young’s modulus. More important could be the shape and surface area versus frictional coefficient. Similarly, this could apply to forces like surface tension, hygroscopic forces and others.

There are other problems to overcome before manufacturing these hooks *en masse,* but it does point to a method of effectively producing magnetic hooks that have been used to conduct electricity commercially as well as heat. Attachment methods are important as well as the way they all come out, which can be distorted from all shape and needs to be considered too. Destructive testing is the only way of assessing this.

This form of data collection and transfer is regarded as another form of mathematics, abstract and applied character. It makes the statement that all members of the set of hooks can be made under the conditions of the microscopy settings given in paper 2 of the set [2]; therefore, a part of the experimentation is already complete. All that is left is manufacture and testing. Only samples that are completely translucent are used, so the intensity of the light is high and able to be differentiated from the background light.

This problem never would have been encountered had the topic been fasteners in general since a straight attachment mechanism would have been sufficiently simple to analyse and model but not manufacture either. It is the mechanical properties that are sought, and the only way of achieving this is to make them from a new material that does not get a lot of attention these days, copper, but through the act of forced self-assembly by applied voltage, it may be possible to arrive at an alternative manufacturing technique or deposition pattern as well as alternative materials such as silicates or gold.
